# A quantitative measure of treatment response in recent‐onset type 1 diabetes

**DOI:** 10.1002/edm2.143

**Published:** 2020-05-14

**Authors:** Brian N. Bundy, Jeffrey P. Krischer, for the Type 1 Diabetes TrialNet Study Group

**Affiliations:** ^1^ Health Informatics Institute University of South Florida Tampa FL USA

**Keywords:** CLINICAL trial, C‐peptide, predictive variable, prognostic variable, type 1 diabetes

## Abstract

**Introduction:**

This paper develops a methodology and defines a measure that can be used to separate subjects that received an experimental therapy into those that benefitted from those that did not in recent‐onset type 1 diabetes. Benefit means a slowing (or arresting) the decline in beta‐cell function over time. The measure can be applied to comparing treatment arms from a clinical trial or to response at the individual level.

**Methods:**

An analysis of covariance model was fitted to the 12‐month area under the curve C‐peptide following a 2‐hour mixed meal tolerance test from 492 individuals enrolled on five TrialNet studies of recent‐onset type 1 diabetes. Significant predictors in the model were age and C‐peptide at study entry. The observed minus the model‐based expected C‐peptide value (quantitative response, QR) is defined to reflect the effect of the therapy.

**Results:**

A comparison of the primary hypothesis test for each study included and a t test of the QR value by treatment group were comparable. The results were also confirmed for a new TrialNet study, independent of the set of studies used to derive the model. With our proposed analytical method and using QR as the end‐point, we conducted simulation studies, to estimate statistical power in detecting a biomarker that expresses differential treatment effect. The QR in its continuous form provided the greatest statistical power when compared to several ways of defining responder/non‐responder using various QR thresholds.

**Conclusions:**

This paper illustrates the use of the QR, as a measure of the magnitude of treatment effect at the aggregate and subject‐level. We show that the QR distribution by treatment group provides a better sense of the treatment effect than simply giving the mean estimates. Using the QR in its continuous form is shown to have higher statistical power in comparison with dichotomized categorization.

## Graphical abstract

This paper proposes a quantitative end‐point in the study of recent‐onset type 1 diabetes that measures the effect of a treatment on the stimulated C‐peptide of an individual patient and, in the aggregate, discriminate those who benefited (responders) from those who did not (non‐responders). The quantitative response measure can be used to evaluate promising biomarkers or other prognostic characteristics and is defined by the difference between the observed and expected C‐peptide levels.

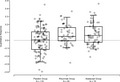

## 1 INTRODUCTION

Researchers have expressed a strong desire to define a measure of response at the individual level in studies of recent‐onset type 1 diabetes (T1D) subjects treated with an experimental agent from a completed randomized clinical trial. Motivation for this reflects a need to provide regulators, considering approval of an experimental therapy, a percentage of subjects that ‘benefited’ from that experimental therapy. As well, patients might better understand the potential benefit of treatment, if put in terms of the chance of achieving a specific state in their disease (eg reduced hypoglycaemic episodes). The desired state should be relevant and meaningful from the perspective of the patient. This paper addresses another purpose: to separate subjects that received the experimental therapy into those that benefitted from those that did not. Benefit for the recently diagnosed type 1 diabetes subject means a slowing (or arresting) the decline in beta‐cell function over time. Serum C‐peptide measured over 2 hours during a mixed meal tolerance test (MMTT) has become an accepted surrogate for beta‐cell function. Presumably, one might conclude that subjects that had minimal decline (or no decline) benefited from the therapy, while those with steeper declines did not. A counterargument is that the rate of decline varies independent of treatment and this variation may stem from differences in the underlying severity of the disease. Thus, it is unclear how to distinguish differential therapeutic benefit from disease severity in the treated group.

Additionally, a goal of biomarker studies is to identify a patient characteristic (usually genetic or immunological) that is associated with (and thus may explain) any variation in therapeutic effect. This association may help elucidate the mechanistic function of the therapy administered or identify a subpopulation for intervention as part of a strategy to refine and develop effective therapies. However, in general, a biomarker that is associated (or statistically speaking, correlated) with C‐peptide decline in the treated group is either *predictive* of benefit or *prognostic* (ie indicative of the severity of T1D). A *prognostic variable* (for recent‐onset T1D) is a characteristic, measured prior to therapy, which correlates with C‐peptide outcome. A key feature is that the correlation is present in both treated and untreated (ie placebo) groups. A *predictive variable* correlates with C‐peptide outcome only for the treated group, and no correlation exists in the placebo group. Thus, an initially promising biomarker requires testing in both placebo and treated samples to distinguish whether it is predictive or prognostic. Age is a classic example of a prognostic variable since there is a strong direct correlation between age and 1‐year C‐peptide decline regardless of the agents that TrialNet has studied to date. This does not preclude the possibility of age being predictive for some experimental agent in the future.

Many attempts at identifying subjects that have benefited from therapy (from those that have not benefited) have dichotomized the change (from baseline) in the stimulated C‐peptide from an MMTT. Responders are often defined to be those above some C‐peptide threshold and the complement being non‐responders. Herold et al^1^, evaluating the effect of anti–CD3‐based response on the change in C‐peptide level, defined as the area under the curve (AUC) mean increase over the fasting C‐peptide level. Response was considered when the value increased by more than 7.5% from the baseline value—7.5% was used because it is one‐half of the C‐peptide interassay coefficient of variation. Mortensen et al^2^ used the coefficients from modelling C‐peptide regressing on HbA1c and insulin dose per kilogram weight to define a responder (if HbA1C per cent +4**∙**insulin dose units per kilogram per 24 hours ≤ 9 then classify as responder). Again Herold et al^3^, evaluating the effect of anti‐CD20, defined response using the coefficient of variation estimate of 0.097. If the 6‐month C‐peptide AUC mean was greater or equal to 90.3 per cent of baseline (≤0.097 decrease), the subject was classified as a responder. In another report by Herold et al^4^, response was defined as <40% decline of C‐peptide at 2 years from baseline. This threshold was selected primarily because all control subjects had ≥40% decline. Beam et al^5^ recommended using strictly no decrease in 6‐month C‐peptide from baseline to define responder. He indicated that the bias (‘the amount by which a responder definition will, on average, over‐ or underestimate the responder percentage in a patient population’) is nearly zero compared with definitions that include some percentage decline (eg 7.5%) as responders where the bias was not negligible. The limitations of these efforts appear ad hoc, possibly data‐driven and are applied to a single study.

This paper proposes the use of an adjusted end‐point we refer to as the quantitative response (QR) and a specific model structure (additive model with an interaction term) for screening biomarkers to determine their predictive or prognostic attribute. The basis of the QR is an analysis of covariance (ANCOVA) model of C‐peptide that adjusts for baseline C‐peptide and age and has been previously described.^6^ The model forecasts the C‐peptide level at 12 months but does not include any effect of an active therapy. The QR is defined as the *observed* 12‐month C‐peptide AUC minus the model's predicted C‐peptide AUC (expected). This age‐adjusted value may reflect a differential benefit of treatment although distinguishing disease severity from a differential treatment effect on a subject‐by‐subject basis is impossible by simply viewing the QR distribution. It requires that a biomarker (eg expressing a mechanistic function of the therapy) be measured and analysed for any association (correlation) with the QR end‐point. The correlation needs to be quantified using an additive model (eg ANCOVA) with QR as the dependent variable and covariates: the biomarker, treatment group (0 = placebo and 1 = active therapy group) and the product of these two covariates (interaction term) as the third covariate. We demonstrate the advantage of the QR and the method of analysis as being general by making various use of six TrialNet studies, the method is not data‐driven (method was not based any biomarker and QR is independent of all but the first five recent‐onset TrialNet studies), and it is based on statistical method that allows expressing the prognostic and/or predictive feature of the biomarker being tested. Our hope is that this approach will provide a uniform and general framework for evaluating biomarkers when the goal is to determine whether the biomarker expresses differential treatment benefit (ie predictive biomarker).

## 2 MATERIALS AND METHODS

### 2.1 Subjects

Baseline and one‐year follow‐up data from five completed TrialNet studies of recent‐onset type 1 diabetes subjects^7^, ^8^, ^9^, ^10^, ^11^ were used in fitting an analysis of covariance (ANCOVA) model. The model cohort is also used to illustrate the utility of the quantitative response measure. The more recently completed antithymocyte globulin (ATG), with and without pegylated granulocyte colony‐stimulating factor (GCSF), trial^12^ was also included to illustrate the generalizability of the proposed statistic on an independent data set. Participants completed a written informed consent and/or assent before participation in these studies. The eligibility for these studies was quite similar in that all had to meet the definition with respect to the diagnosis of type 1 diabetes and enrolment within 100 days of diagnosis and a C‐peptide level ≥ 0.2 pmol/mL. The studies did vary at the younger age range by design with an upper limit of 45 years.

### 2.2 Statistical considerations

The primary outcome is the C‐peptide levels from the first 2 hours of a mixed‐meal tolerance test (MMTT). The trapezoidal rule is applied to the five timed measurements and then summed to approximate the area under the curve then divided by the 120‐minute interval, henceforth C‐peptide AUC mean.

#### 2.2.1 Model

In our previous paper (Bundy & Krischer, 2016), we fit the ANCOVA model to the 1‐year C‐peptide AUC mean from the modelled cohort regressing on age at study entry, natural log‐transformed (after adding 1) baseline C‐peptide AUC mean and each experimental treatment assignment. Although there was no systematic process in considering other covariates, neither body mass index (transformed to z‐score) nor the second‐degree term for age (which allows a parabolic fit to transformed C‐peptide value) provided an improved model fit. HbA1c was statistically significant but because of collinearity with baseline C‐peptide the improvement in the model fit was negligible (*R* ^2^ increased from .593 to .599). The following equation (from the fitted model) gives the predicted transformed 1‐year C‐peptide AUC mean given the age and baseline C‐peptide of any subject if administered placebo:

(1) $$E\left[\ln({\text{Cp}}_{\text{1year}}+1)\right]=-0.191+0.812\cdot \ln\left({\text{Cp}}_{0}+1\right)+0.00638\cdot \text{Age}$$

The Cp variables represent the pertinent C‐peptide AUC means, and Age is the year of age at study entry. ln is the natural logarithm function, and *E*[·] represents the expected value. The square root of the residual mean squared error (RMSE) is 0.151.

#### 2.2.2 Quantitative response (QR)

Having measured the 1‐year C‐peptide AUC mean of a subject (usual units: nano‐moles per litre; the time units in minutes are cancelled out by division by 120 minutes), we can compute the transformed difference between their observed C‐peptide sAUC from their expected level (ie observed minus expected). Hence, for an individual, *i*, the QR is defined to be

(2) $${\text{QR}}_{i}=\ln\left({\text{Cp}}_{\text{1year,}i}+1\right)-0.812\cdot \ln\left({\text{Cp}}_{0,i}+1\right)-0.00638\cdot {\text{Age}}_{i}+0.191$$

(the units: plus‐one‐natural‐log of nano‐moles per litre). It may be useful to consider QR as an adjusted normalized value of the 1‐year C‐peptide with the empirical distribution centred at zero in the absence of any treatment effect.

To evaluate the variation of the QR as a function of the observed baseline C‐peptide (statistically referred to as heteroscedasticity), we followed White's method.^13^ A linear regression model was fitted to the squared QR values regressing on baseline C‐peptide to estimate the change in variance by baseline C‐peptide. The fit indicated that the median square root of the RMSE was 0.152 (10th and 90th percentiles: 0.108 and 0.199). Although the variance of the QR varies with baseline C‐peptide, the QR is an accurate estimate (the statistical term is unbiased estimate. A non‐technical explanation is if the QR could be theoretically measured multiple times on the same subject, the average of those multiple values would be ever nearer the true QR value). The variance of QR was not correlated with age. (An analysis demonstrating that the QR is independent of age and baseline C‐peptide can be found in Appendix S1: Figures S1 and S2).

#### 2.2.3 Estimating statistical power

Monte Carlo simulation was employed to estimate the statistical power when testing for a correlation between a linear predictive variable (ie biomarker) and QR. Each simulation sampled baseline C‐peptide and age pairs from the modelled cohort at random with replacement (real data). The sample size for each simulated trial was set to typical size TrialNet phase II recent‐onset trial (ie 33 and 17 subjects in experimental and placebo group, respectively, having the end‐point of 1‐year C‐peptide AUC mean). The size was based on 50% minimal detectable difference (MDD; algebraically: Δ) in the treatment group means at 12 months poststudy entry (0.376 vs. 0.564 nano‐moles/L), 85% statistical power, type 1 error of 0.05 (1‐tail test), allocation ratio of 2:1 (experimental: placebo) and standard deviation of 0.158 (see Bundy & Krischer). The predictive biomarker was formulated such that it had a range from zero to twice the mean (to preserve symmetry) when following a normal distribution. The treatment effect varied linearly with the biomarker, that is, Δ∙*biomarker*. For generalizability, two other distributions for the predictive variable were considered: the chi‐square and uniform distributions. The mean treatment effect for all three biomarker distributions was set at 1.18Δ. This value represents midway between the MDD and the largest treatment effect seen in a TrialNet study of recent‐onset type 1 diabetes (the ATG/GCSF study had an effect of 1.37Δ).^12^ For the normal and uniform distributions, the treatment effect ranged from 0 to 2.36Δ. The right‐skewed square root of the chi‐square distribution function required extending the range to 4.25 and also setting the degrees of freedom to 1.815 in order to keep the mean the same as the other distributions. For the normal distribution, the standard deviation was set to 1.18/2; extreme values outside the range of (0, 2.36) were resampled for both the normal and the chi‐square distributions. To reflect biomarker measurement error (unexplained variation), an independent normally distributed random variables with mean 0 and variances of *σ* ^2^ and *σ* ^2^/2 were added to the predicted value. The *σ* ^2^ is the unexplained variance of the QR (or C‐peptide AUC mean after adjustment) which was estimated as 0.151 from the modelled cohort.

The choice of the biomarker measurement error is somewhat arbitrary but considered to be in the same range as QR measurement error. The primary purpose is to illustrate the loss in power for some response definitions. We did not consider a dichotomized predictive variable for the sake of simplicity. In general, considerably larger sample size is required to have nominal power in detecting a dichotomous predictive variable. We set the simulations to 20 000 replications, which yields a maximum 95% confidence interval for the statistical power of ±0.00693 (binomial approximation).

A formal statistical test was employed to each simulated trial with a threshold of significance set at 0.05 (one‐sided). The test was based on a three covariate ANCOVA model of QR: biomarker, treatment group (parametrized: 0 = placebo group and 1 = experimental treatment group) and the product (interaction) term; the coefficient of this last term was the basis of the test.

#### 2.2.4 Percentile‐based responder

A series of QR thresholds were used to illustrate a forced dichotomized responder definitions to be evaluated in the simulation. The goal was to determine what QR threshold might be preferable, if one required classifying subjects as responders and non‐responders on the basis of the QR. The control group was used to establish any shift in the QR distribution by calculating the mean and the average dispersion for each treatment group (pooled variance). The percentiles extracted from the normal distribution were used as thresholds to classify responders in the treated group. The logic of this algorithm is that it corrects the thresholds for a trial where the QR distribution is not centred at zero (this may happen when the eligibility criteria have been altered or the experimental agent being studied has altered the subject selection process).

All analyses were conducted in TIBCO Spotfire S+™ 8.2 Workbench.

## 3 RESULTS

**Figure 1:**
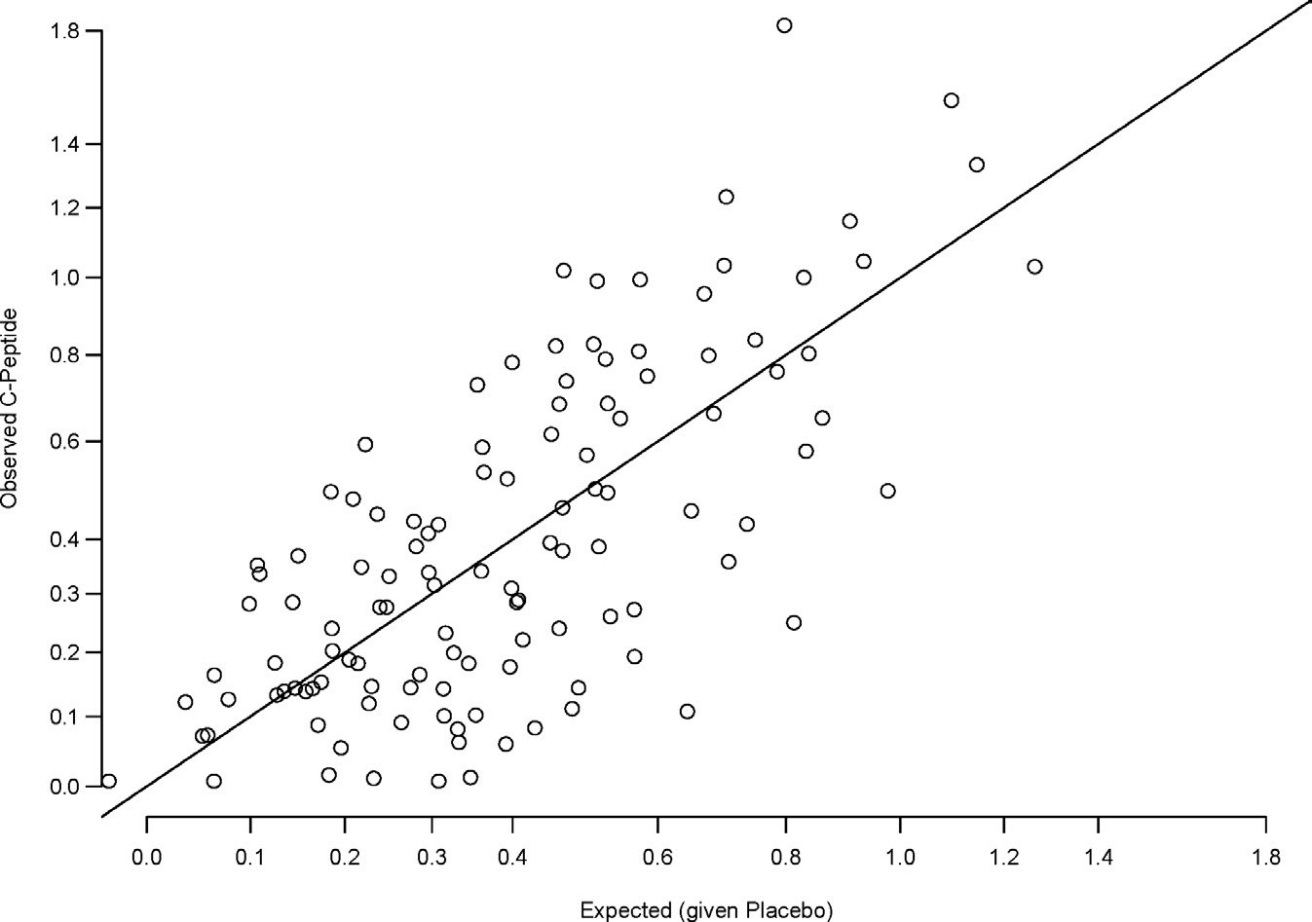
Observed 1‐year C‐peptide by expected model‐based C‐peptide for subjects in the placebo groups. The vertical distance from the point to the diagonal line is the observed 1‐year C‐peptide minus the model‐based expected 1‐year C‐peptide, that is, the quantitative response (QR)

The *expected* C‐peptide level at 1 year for a subject is calculated by substituting their age at entry, and their baseline C‐peptide value in Equation 1. Figure 1 displays the *observed* transformed 1‐year C‐peptide and the corresponding *expected* C‐peptide for all placebo group subjects from the modelled cohort. The figure provides a visual of the *observed* minus *expected* distribution via the vertical distances from the diagonal line to each point (the QR is negative if the point is below the diagonal line). There are 60 (50.8%) positive and 58 negative QRs indicating symmetry around zero overall, and the symmetry is reasonably consistent across the range of *expected* baseline C‐peptide values. The variation of the QR was greater for greater values of *expected* C‐peptide. No such correlation was present for age. A regression line adequately expresses the variation in QR for the range of baseline C‐peptide. The median variation was 0.152, and the 10th and 90th percentiles were 0.108 and 0.199, respectively. Regardless of this change in the QR variation, the QR value is accurate (unbiased estimator).

**Figure 2:**
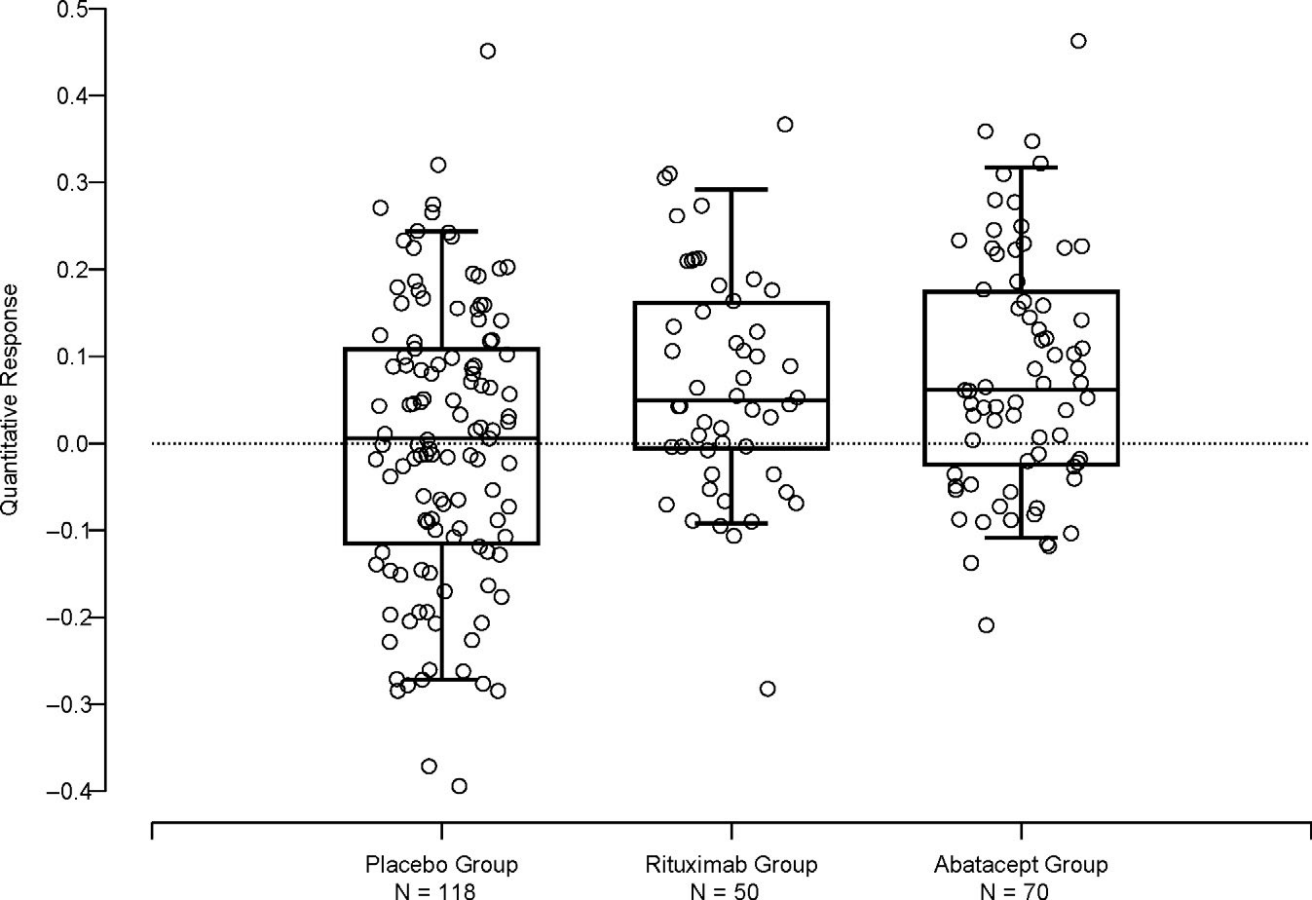
Box plot of quantitative response of three treatment groups: Placebo [from the combined studies], rituximab^8^ and abatacept.^10^ The horizontal sides of the box mark the 25th and 75th percentiles, the horizontal line within the box marks the median, and the whiskers mark the 5th and 95th percentiles. The horizontal variation of the points is for visual clarity and has no meaning

Figure 2 is a boxplot of the QRs for three treatment groups and displays all observations. The symmetry of QRs around zero is reaffirmed in the placebo group. In contrast, there is a positive shift in the QR distribution for the experimental treatment groups compared with the placebo group from these two trials (TrialNet rituximab and abatacept studies).^8^, ^10^ There are 34 (68.0%) and 48 (68.6%) subjects with QRs greater than zero in the rituximab‐ and abatacept‐treated groups, respectively. The median QR is an estimate of the treatment effect albeit on a scale which is not relatable clinically. We suggest that displaying the QRs for every subject by treatment group provides a better sense of the treatment effect than simply the average, specifically, that there may be a fair number of subjects in the treated group with lower QRs than the control group despite an overall positive result.

The QR may be used to test a treatment effect using a simple t test, (or a non‐parametric test such as the Wilcoxon rank‐sum test). Table 1 displays a comparison of the recomputed primary hypothesis test (any slight difference from published results is due to post‐publication data corrections) and a t test of the QR value by treatment group. The table includes the treatment effect coefficient from the analysis of covariance model adjusting for age and baseline C‐peptide and the Wald significance level (the stipulated primary hypothesis test used in the primary analysis of each study). For comparison, the mean difference in the QR between treatment groups and the significance level of the associated t test are provided. The right side of the table is the comparison of the second experimental treatment to the control group for the three TrialNet studies that randomized to three treatments. The two analytical approaches for each comparison agree. The TrialNet ATG/GCSF study^12^ is independent of the data used in determining the QR coefficients [Equation 2]; yet, the two approaches agree for both experimental groups.

**Table 1:** Treatment effect estimates and significance levels for six TrialNet studies by the original primary hypothesis test (Wald test for treatment from the ANCOVA model) and two‐sample t test of quantitative response (QR)

Trial^a^	Experimental Group vs. Placebo				Second Experimental Group vs. Placebo			
	ANCOVA Model		Quantitative Response t test		ANCOVA Model		Quantitative Response t test	
	Treatment Effect	*P*‐value	Treatment Effect	*P*‐value	Treatment Effect	*P*‐value	Treatment Effect	*P*‐value
DZB & MMF^7^	0.0244	.24	0.00765	.43	0.0123	.37	0.0266	.22
Rituximab^8^	0.0637	.03	0.0668	.03	–	–	–	–
GAD + Alum^9^	−0.0206	.74	−0.00556	.57	−0.00121	.52	−0.0151	.69
Abatacept^10^	0.0803	.006	0.0794	.009	–	–	–	–
Canakinumab^11^	−0.0068	.57	0.0042	.46	–	–	–	–
ATG + GCSF ^12^	0.157	<.001	0.159	<.001	0.0829	.03	0.0832	.03

^a^ DZB & MMF: mycophenolate mofetil (MMF) with or without daclizumab (DZB),^7^ rituximab,^8^ GAD + Alum: recombinant human glutamic acid decarboxylase (GAD) formulated in aluminium hydroxide (GAD‐Alum),^9^ abatacept,^10^ canakinumab^11^ and antithymocyte globulin (ATG) and pegylated granulocyte colony‐stimulating factor (GCSF).^12^

**Figure 3:**
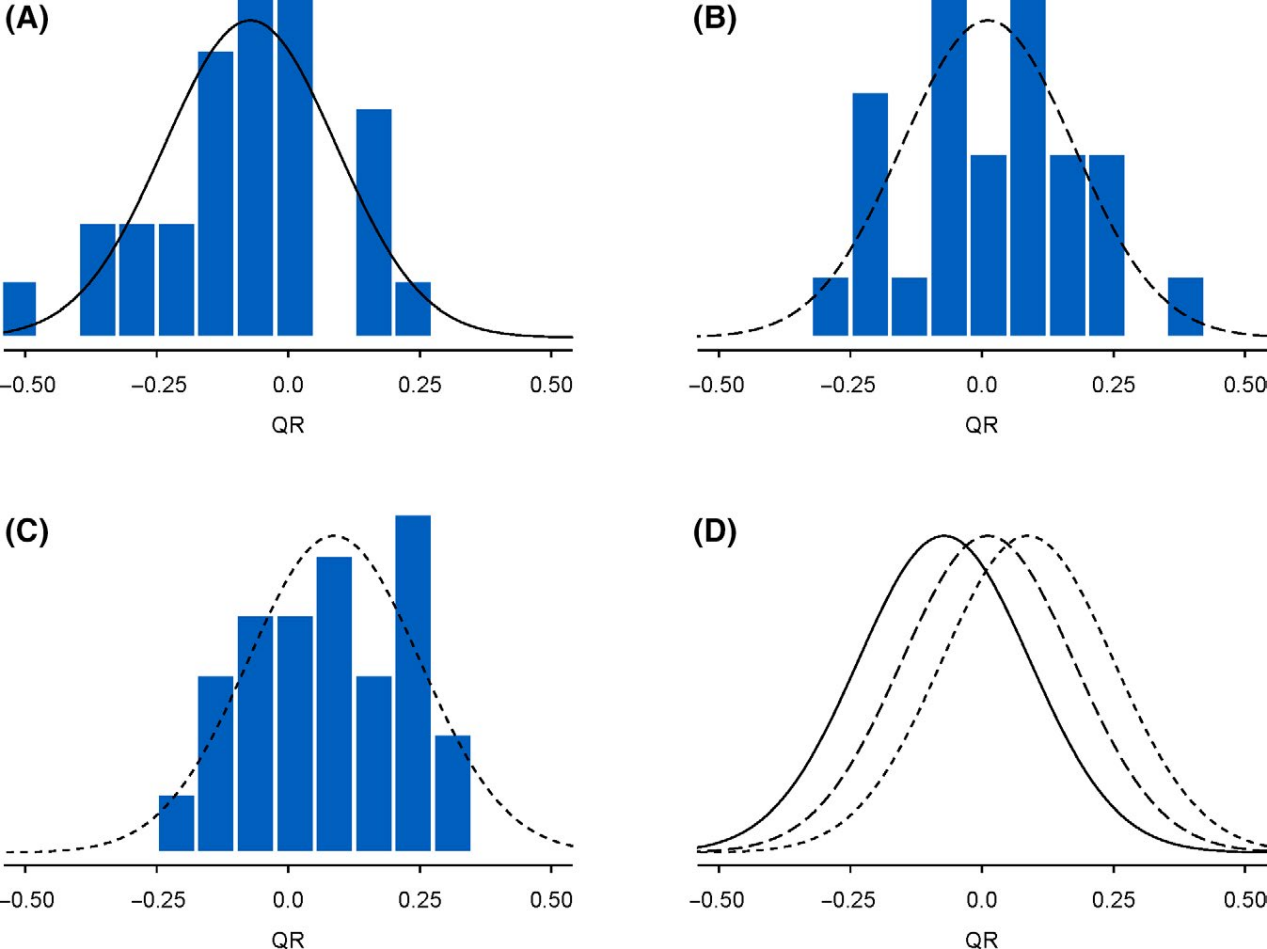
Quantitative response histogram by treatment group of TrialNet ATG/GCSF study: panel A is the placebo group, panel B is the ATG and GCSF group, and panel C is the ATG‐only group. Superimposed on each histogram is the normal density function where the mean was set to the mean of the quantitative response of the group and the standard deviation was set to the pooled (over the 3 groups) standard deviation of the quantitative response. Panel D displays just the three treatment group normal density functions using the same line type as in panels A‐C, respectively, solid = placebo, short dash = ATG only and long dash = ATG and GCSF

A histogram of the QRs for each treatment group from TrialNet ATG/GCSF study is in Figure 3. Also displayed are the normal bell‐shaped curve (probability density) determined from the average QR of each group and an overall variation of QR (pooled variance). It is visually apparent that there is an increase in the QRs in the ATG‐only group compared with the placebo group and the treatment effect appears to be a positive shift for the entire group rather than for just certain levels of QR’s.

**Figure 4:**
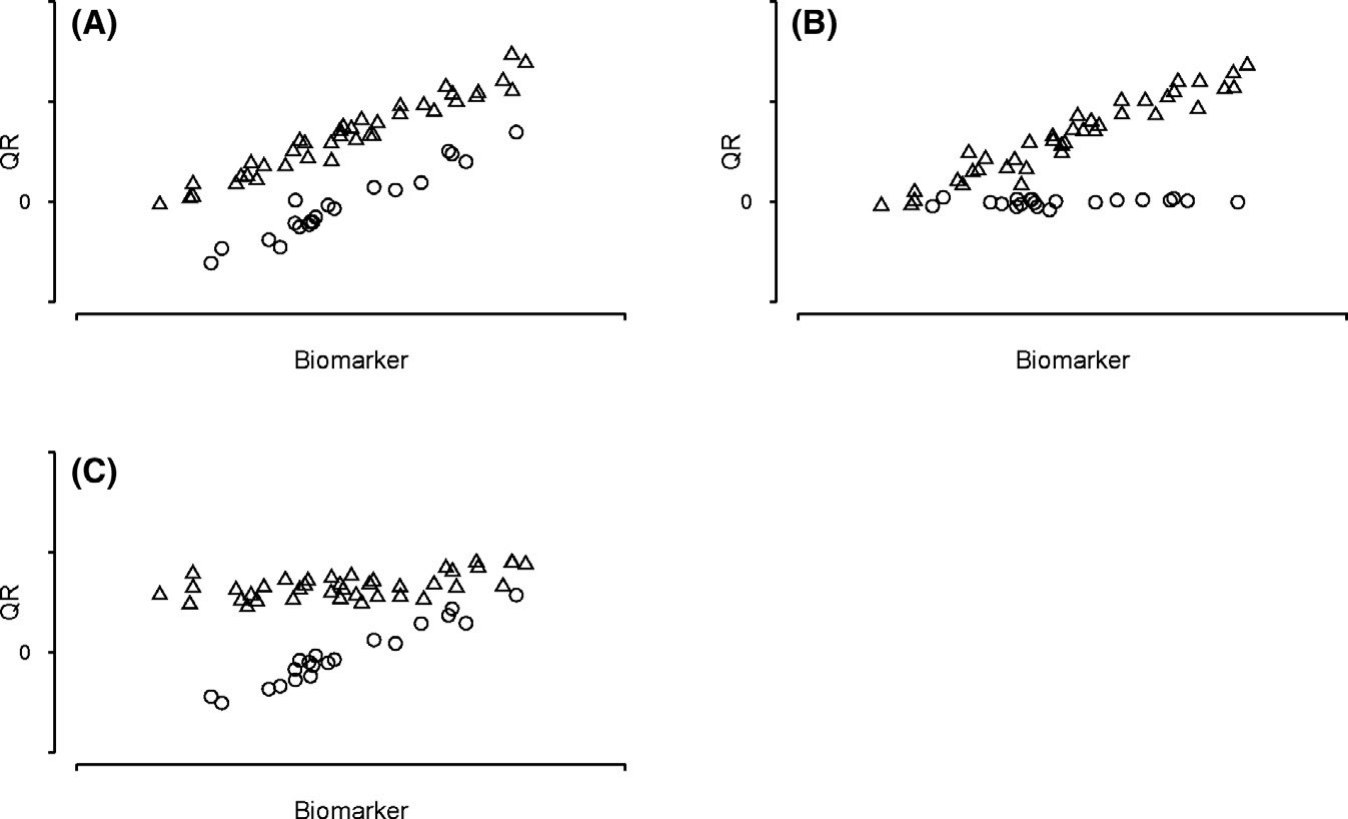
Scatterplots of the relationship of three types of hypothetical biomarkers with treatment group and QR. Panel A: prognostic biomarker, Panel B: predictive biomarker, Panel C: both prognostic and predictive biomarker. Other relationship other than these three may exist. The variation around the regression line (not shown) has been set low for visual clarity

We propose an analytical method to screen biomarkers to assess their prognostic or predictive attribute. Fit a statistical model of QR (dependent variable) with three covariates (independent variables): the biomarker to be evaluated, the treatment group category (both treatment groups: 0 = placebo and 1 = treatment) and an interaction term of the biomarker and treatment covariates. The fitted coefficients and their significance levels will delineate the biomarker as prognostic, predictive, both or neither. Figure 4 provides scatterplots of QR and three hypothetical biomarkers representing three possible relationships with treatment and the end‐point QR. These hypothetical biomarkers represent prognostic, predictive and both in panels A, B and C, respectively. All three figures reflect an active experimental therapy. In addition, panels A and B display a positive association between the biomarker and QR. Panel C displays a negative association between the biomarker and treatment effect but a positive association between the biomarker and QR for the placebo group only. Other relationships are possible.

We evaluated the chance of detecting (statistical power) a predictive biomarker using the model structure described above and a formal statistical test associated with the interaction term. The simulation studies set the treatment effect to be proportional to the value of the biomarker (linear effect). Measurement errors were included for both the QR (via C‐peptide AUC mean) and the biomarker to reflect the reality of measuring constituents from subject samples. Although we do not suggest strict adherence to 0.05 significance level when evaluating biomarkers (lack of statistical power is often an issue when testing interaction terms) for their predictive attribute, we did so for the simulations. Table 2 presents the chance of detecting (statistical power) a predictive biomarker for two possible biomarker measurements errors, several QR‐based responder definitions and QR as a continuous variable. The QR‐based responder definitions were determined by the thresholds from the percentiles of the normal distribution (in 5% increments) where the mean was set to the QR control group mean and the variation (variance) was the combined variation from both treatment groups (pooled variance).

**Table 2:** Statistical power (via simulation) to detect a predictive biomarker by several responder definitions (and QR) and two measurement errors of the biomarker. The treatment effect is proportional to the biomarker (minimum of no effect and a maximum of 2.36Δ)

Responder definition	Measurement error^b^	
	*σ*	*σ*/2
≥55th Percentile	0.309	0.324
≥60th Percentile	0.335	0.351
≥65th Percentile	0.35	0.368
≥70th Percentile	0.352	0.374
≥75th Percentile	0.338	0.36
≥80th Percentile	0.295	0.31
≥85th Percentile	0.225	0.24
≥90th Percentile	0.131	0.141
≥95th Percentile	0.0486	0.048
None (Continuous)	0.587	0.616

^a^ The normal distributed biomarker was restricted such that 0 ≤ *biomarker (b)* ≤2.36 (symmetry retained) and the treatment effect was expressed as *b*·Δ where Δ = 0.182.

^b^ The standard deviation of the measurement errors of the biomarker is expressed as fractions of *σ*, the unexplained standard deviation of QR, 0.151.

The continuous QR produced the highest chance of detecting a predictive biomarker (statistical power) regardless of the variation in measuring the biomarker (measurement error). This was true with other levels of variation and when the biomarker distribution was not bell‐shaped (see Appendix S1: Table S1). The chance of detecting a predictive biomarker (statistical power) when dichotomizing the QR for the various percentile‐based responder definitions varied considerably. From Table 2, the highest chance of detecting a predictive biomarker occurs at the 70th percentile definition of responder regardless of the precision of measuring the biomarker (measurement error). It also remains the maximum chance of detecting (greatest statistical power) when the biomarker distribution is other than bell‐shaped (see Appendix S1: Table S1). Nonetheless, the continuous QR provides the maximum chance of detecting a predictive biomarker over any of these responder definitions. When relaxing the level of significance from 0.05 to 0.10 (offered as a solution when testing interactions and statistical power is low), the chance of detecting a predictive biomarker when analysed as a continuous variable comes close to conventional levels (0.720 and 0.746 when the measurement error of the biomarker is *σ* and *σ*/2, respectively. See Appendix S1: Table S2).

## 4 DISCUSSION

We have shown that the analysis of covariance (ANCOVA) model of 1‐year log‐transformed, age‐adjusted, C‐peptide is consistently good predictor across several TrialNet studies. We defined the quantitative response (QR) as the *observed* 1‐year C‐peptide minus the model‐based *expected* C‐peptide level. We confirmed the excellent behaviour of QR using a few of the studies used in fitting the model as well as a trial that was independent of the modelling.^12^ Defined in this way, a positive shift in the QR distribution provides the magnitude of the treatment effect on C‐peptide for an ‘active’ treatment, while the QR mean is around zero for the placebo group (or an inactive treatment). The QR is calculated at the subject level and so provides a visually informative way of viewing all subjects in the study and the treatment effect. In addition, the QR allows for a simple analytical test of treatment effect consistent with the standard ANCOVA model test.

Without any a priori biological basis, there is little justification for choosing any particular threshold to partition the treated group into distinct categories of responder and non‐responder. This is particularly true if the interpretation is to identify subjects that had treatment benefit from those that did not (or benefited minimally). We offer several arguments to support this contention. One, some of the individuals classified as ‘responders’ may have attained their QR value (or less C‐peptide decline) because they have inherently less severe disease (without considering the benefit they received from treatment). Two, when setting the threshold at a very stringent level (eg no C‐peptide decline), those classified as ‘responders’ have a higher likelihood of having exaggerated levels of C‐peptide. This is a proven statistical phenomenon referred to as regression to the mean. Three, it is possible that subjects that were destined to have low C‐peptide levels if not treated, had substantial benefit due to treatment but still not greater than the selected threshold used to define responder. Thus, the degree of misclassification due to dichotomizing may be substantial and misclassification may go either way. Our simulation studies clearly indicate that using the QR in its continuous form will increase the chance of discovering a biomarker correlated with treatment effect, that is, a predictive biomarker.

Nonetheless, if a compelling reason remains to group subjects as responders and non‐responders, then using the placebo group's 70th or 65th percentile of the QR distribution as a threshold to partition the experimental treatment group into responder categories provides the smallest reduction in the chance of detecting a predictive biomarker. We suggest using the placebo or control group to define the threshold and did so in our simulations. Not presented were other ways of determining a QR threshold to define response. The power was slightly less (2 to 3%) than the values displayed in Table 2 when using the empirical percentiles of QR from the placebo group. Alternatively, using a fixed percentile threshold taken from the normal distribution with mean of zero and standard deviation of 0.151 (determined from the modelled cohort) provided a slightly higher statistical power than in Table 2 but only by less than 1%. However, this fixed threshold ignores the QR distribution from the placebo group of the trial analysed. Determining thresholds using the QR distribution from the internal control group adjusts for any possible shift that may occur in a future trial.

It is imperative that investigators involved in the analysis of biomarkers in the context of a clinical trial understand the difference between a prognostic and a predictive biomarker. It is essential that any biomarker that is correlated with QR in the treated group be evaluated in the placebo group. We suggest to model the QR with two covariates, treatment group and the biomarker to be evaluated, as well as an interaction term; this allows a way to quantify the predictive from the prognostic effect of the biomarker. The interpretation of the biomarker's utility will be dramatically different. If a biomarker is prognostic, it will be advisable to measure this marker in subsequent trials in order to adjust for it in the analysis. If the biomarker is predictive, it will likely have value for targeting subjects for further study, particularly in primary prevention trials of the agent associated with the biomarker.

Our analytical approach provides less statistical power (see Table 2) than approaches that ignore the distinction between predictive and prognostic biomarkers. Testing for an interaction effect term in any model is always subject to less power than the main effect terms. Testing at a relaxed level of significance (α = 0.10) increases the statistical power to an acceptable level. While rectifying the low power, such an adjustment increases the chance of a false‐positive result. In addition, these studies usually evaluate multiple biomarkers (multiple testing problem) that further contributes to the risk of one or more false positives. In dealing with both circumstances, a reasonable strategy would be not to adjust for multiple tests but rather consider the detection of any predictive biomarker as hypothesis generating and pursue confirmation in an independent setting. One weakness of the QR is that there remains a fair amount of unexplained variance. We have suggested that using our method may identify a biomarker as predictive. Alternatively, our approach could identify a biomarker as prognostic and therefore lead to a revised QR equation which would have reduced unexplained variance, that is, improved prediction at the subject level. The QR units are not meaningful to investigators except that a positive value represents a subject that had higher 1‐year c‐peptide level than what was expected from their age and baseline c‐peptide, and conversely, a negative QR means lower 1‐year c‐peptide than expected. One caution is in order, if an agent provides differential effect across age, then using the QR method may not be prudent at least until one quantifies its predictive effect. For this reason, we suggest checking age as a predictive variable using the observed change in c‐peptide and then proceed using age‐adjusted QR method if there is no convincing evidence of such an effect.

Future plans are to apply the QR method to empirical data in which biomarkers have been measured from the samples in both treated and controls. Furthermore, to address the timing of when the biomarker was measured (baseline and after/during treatment), this timing not only changes the interpretation of the results but also changes the analytical methods employed.

## CONFLICT OF INTEREST

The authors declare that they have no conflicts of interest. Drs. Bundy and Krischer work at the TrialNet Coordinating Center funded by the NIDDK of the NIH.

## AUTHOR CONTRIBUTIONS

Brian Bundy and JP Krischer researched data, contributed to discussion, wrote the manuscript and reviewed/edited the manuscript.

## ETHICAL APPROVAL

All TrialNet protocols included in this study were approved by each participating institution's Institutional Review Board (IRB) or Ethics Committee/Research Ethics Board (EC/REB). The NIH (National Institute of Diabetes and Digestive and Kidney Diseases) was the sponsor for these trials.

## Supporting information

Supporting information

Appendix S1

## Data Availability

All TrialNet data generated or analysed during this study can be requested from the NIDDK Central Repository at https://repository.niddk.nih.gov/studies/trialnet/.
