# Supplementary material for: A quantitative measure of treatment response in recent‐onset type 1 diabetes
Source: Endocrinol Diabetes Metab. 2020 May 14;3(3):e00143. doi: 10.1002/edm2.143 (PMC7375065; doi:10.1002/edm2.143)
Supplement: Supplementary file 1 — Supporting information [file EDM2-3-e00143-s001.docx]

**Supplement to: A Quantitative Measure of Treatment Response in Recent Onset Type 1 Diabetes**

**Independence of QR:** The reviewers requested a scatterplot of the QR by age and separately by baseline c-peptide to affirm that the QR is independent of these variables. Figures S1 and S2 provide the visual support of that independence. A simple linear regression was used to verify that neither covariate was correlated with QR.

**Figure S1: Scatterplot of QR by age at Entry for the cohort on which the model was built. The Wald test from the simple linear regression of QR regressing on age was not significant (p = 0.67).**

**Figure S2: Scatterplot of QR by baseline C-peptide for the cohort on which the model was built. The Wald test from the simple linear regression of QR regressing on baseline c‑peptide was not significant (p = 0.77).**

**Statistical Power:** Additional simulation studies were conducted when the biomarker, and hence, the treatment effect was not normally distributed. Table S1 provides the statistical power estimates for two other distributions and repeats the normal distributed biomarker results for comparison. The 65^th^ - 70^th^ percentile definition for response yields the largest statistical power for the all combinations of the biomarker distribution and measurement error variance.

**Appendix Table S1: Statistical power to detect a predictive biomarker based on simulations at the α = 0.05. The treatment effect is a function of the biomarker. The table rows reflect various ways to categorize the QR into responder and non-responder prior to analysis. The minor columns reflect two possible levels of unexplained measurement error of the biomarker and the major columns reflect three distributions of the biomarker. For comparison, table 1 in the main body of the article is repeated.**

| Categorization of QR | Biomarker Distribution  (statistical representation and general description) | | | | | |
| --- | --- | --- | --- | --- | --- | --- |
|  | $\sqrt{\chi_{(d.f.=1.815)}^{2}}$ Right-skewed* | | $Normal(\mu=1.18,\sigma=1.18/2)$ Bell-shaped* | | $Uniform(0,2.36)$ Flat* | |
|  | Measurement Error# | | Measurement# | | Measurement Error# | |
|  | $\sigma$ | $\frac{\sigma}{2}$ | $\sigma$ | $\frac{\sigma}{2}$ | $\sigma$ | $\frac{\sigma}{2}$ |
| 55th Percentile | 0.309 | 0.324 | 0.362 | 0.378 | 0.445 | 0.449 |
| 60th Percentile | 0.335 | 0.351 | 0.397 | 0.412 | 0.482 | 0.490 |
| 65th Percentile | 0.35 | 0.368 | 0.423 | 0.439 | 0.500 | 0.509 |
| 70th Percentile | 0.352 | 0.374 | 0.427 | 0.448 | 0.502 | 0.511 |
| 75th Percentile | 0.338 | 0.360 | 0.411 | 0.429 | 0.473 | 0.482 |
| 80th Percentile | 0.295 | 0.310 | 0.356 | 0.373 | 0.408 | 0.413 |
| 85th Percentile | 0.225 | 0.240 | 0.26 | 0.270 | 0.302 | 0.301 |
| 90th Percentile | 0.131 | 0.141 | 0.145 | 0.148 | 0.167 | 0.163 |
| 95th Percentile | 0.0486 | 0.048 | 0.052 | 0.0505 | 0.0544 | 0.0552 |
| Continuous | 0.587 | 0.616 | 0.747 | 0.767 | 0.805 | 0.820 |

* The range of the normal distribution was restricted as 0 ≤ *biomarker* ≤ 2.36 (symmetry retained). For the square-root of the chi-square ($\chi_{(d.f.=1.815)}^{2}$) distribution the maximum was restricted as: 0 ≤ *biomarker* ≤ 4.25. The uniform distribution provides equal probability that *m* is any value within the range: 0 to 2.36.

# The standard deviation of the measurement errors of the biomarker selected are expressed as fractions of $\sigma$, the unexplained standard deviation of QR, 0.151

**Appendix Table S2: Statistical power to detect a predictive biomarker based on simulations at the α = 0.10. The treatment effect is a function of the biomarker. The table rows reflect various ways to categorize the QR into responder and non-responder prior to analysis. The minor columns reflect two possible levels of unexplained measurement error of the biomarker and the major columns reflect three distributions of the biomarker. For comparison, table 1 in the main body of the article is repeated.**

| Categorization of QR | Biomarker Distribution  (statistical representation and general description) | | | | | |
| --- | --- | --- | --- | --- | --- | --- |
|  | $\sqrt{\chi_{(d.f.=1.815)}^{2}}$ Right-skewed* | | $Normal(\mu=1.18,\sigma=1.18/2)$ Bell-shaped* | | $Uniform(0,2.36)$ Flat* | |
|  | Measurement Error# | | Measurement# | | Measurement Error# | |
|  | $\sigma$ | $\frac{\sigma}{2}$ | $\sigma$ | $\frac{\sigma}{2}$ | $\sigma$ | $\frac{\sigma}{2}$ |
| 55th Percentile | 0.482 | 0.504 | 0.552 | 0.564 | 0.636 | 0.645 |
| 60th Percentile | 0.507 | 0.524 | 0.582 | 0.603 | 0.657 | 0.669 |
| 65th Percentile | 0.513 | 0.533 | 0.598 | 0.618 | 0.670 | 0.680 |
| 70th Percentile | 0.512 | 0.530 | 0.601 | 0.622 | 0.659 | 0.671 |
| 75th Percentile | 0.495 | 0.513 | 0.583 | 0.609 | 0.628 | 0.642 |
| 80th Percentile | 0.453 | 0.469 | 0.534 | 0.553 | 0.559 | 0.568 |
| 85th Percentile | 0.369 | 0.388 | 0.435 | 0.456 | 0.446 | 0.451 |
| 90th Percentile | 0.252 | 0.267 | 0.292 | 0.297 | 0.285 | 0.292 |
| 95th Percentile | 0.111 | 0.120 | 0.122 | 0.126 | 0.113 | 0.114 |
| Continuous | 0.720 | 0.746 | 0.847 | 0.861 | 0.888 | 0.896 |

* The range of the normal distribution was restricted as 0 ≤ *biomarker* ≤ 2.36 (symmetry retained). For the square-root of the chi-square ($\chi_{(d.f.=1.815)}^{2}$) distribution the maximum was restricted as: 0 ≤ *biomarker* ≤ 4.25. The uniform distribution provides equal probability that *m* is any value within the range: 0 to 2.36.

# The standard deviation of the measurement errors of the biomarker selected are expressed as fractions of $\sigma$, the unexplained standard deviation of QR, 0.151

**Responder Thresholds:** The implications of selecting a threshold to define responders maybe visualized by a reverse cumulative distribution function (rCDF) of the outcome variable e.g., C‑peptide decline. Based on our simulation studies considering a threshold for QR is likely a better choice than an unadjusted/untransformed outcome. The percent of subjects (for each group) to be classified as a responder for any threshold is illustrate in Figure S3 for TrialNet’s Abatacept study (TN-09). The y-axis provides the percent of subjects classified as responders as a function of QR threshold. Most of the responder definitions in the literature tend to select minimal C-peptide loss as a threshold. We conjecture two explanations for this tendency. One, to keep to a reasonable minimum the number of placebo subjects classified as responders, which is a senseless designation. Two, ostensibly those treated with the active therapy experiencing no decline in their c-peptide benefited from the therapy more than those with greater declines in c-peptide but also treated. The later reason may not be true because we do not know what decline a treated subject would have had if not treated. A treated subject’s decline may be more the product of the severity of their disease relative to another treated subject than a differential effect of the therapy.

Consider for example the threshold of 0.210, it classifies as responders only 9.7% in the placebo group but classifies 21% in the Abatacept group; a difference of 13.2%. The maximum difference of 25.6% occurs when using a threshold of 0.024. Suppose that Abatacept provided the same benefit for every subject on the QR scale. For such a scenario, the rCDF of the treated group would be shifted to the right by 0.086 (the QR group mean difference of the Abatacept trial). Taking two normal distribution and setting the parameters to the two group means and common variance of the Abatacept trial provides an idealized rCDF’s displayed in Figure S4. The largest difference between groups of 23% is at the threshold of 0.037 (the midpoint between the two normal distribution means). These idealized estimates are similar to the empirical estimates of the Abatacept trial, 0.024 and 25.6%, respectively. Moreover, the general shape of the empirical and the theoretical rCDF’s are similar. This is far from sufficient evidence of equal benefit among the treated subjects but it cannot, and should not, be ruled out.

**Figure S3: Percent of Subjects (“Responders”) as a Function of quantitative response Threshold for Placebo and Abatacept treatment groups [7]. The maximum percent difference of 25.6% at a quantitative response of +0.024.**

**Figure S4: Hypothetical reverse cumulative distribution function of the quantitative response (QR) using a normal distribution. The means and standard deviation for the normal distributions are from the same data displayed in Figure S3 (TrialNet Abatacept trial [7]). The mean of the hypothetical placebo group is -0.0054 and the mean of the active treatment group is 0.080. This figure provides the hypothetical percent of responders as a function of the QR threshold for each treatment group. The maximum percent difference of 23.3% is at the quantitative response threshold of 0.037 (average of the two means).**
