# Supplementary material for: A quantitative measure of treatment response in recent‐onset type 1 diabetes
Source: Endocrinol Diabetes Metab. 2020 May 14;3(3):e00143. doi: 10.1002/edm2.143 (PMC7375065; doi:10.1002/edm2.143)
Supplement: Supplementary file 2 — Appendix S1 [file EDM2-3-e00143-s002.pdf]

ONLINE APPENDIX. Type 1 Diabetes TrialNet Study Group.
Personnel as of 1/28/2019.

Steering Committee: Carla J. Greenbaum (Benaroya Research Institute), Mark A. Atkinson (University of Florida), David A. Baidal (University of Miami), Manuela Battaglia (San Raffaele University), Dorothy Becker (University of Pittsburgh), Penelope Bingley (University of Bristol), Emanuele Bosi (San Raffaele University), Jane Buckner (Benaroya Research Institute), Mark Clements (The Children's Mercy Hospital), Peter G. Colman (Walter & Eliza Hall Institute of Medical Research), Linda DiMeglio (Indiana University), Carmella Evans-Molina (Indiana University), Stephen E. Gitelman (University of California, San Francisco), Robin Goland (Columbia University), Peter Gottlieb (Barbara Davis Center for Childhood Diabetes), Kevan Herold (Yale University), Mikael Knip (University of Helsinki), Jeffrey P. Krischer (University of South Florida), Ake Lernmark (Skane University Hospital), Wayne Moore (The Children's Mercy Hospital), Antoinette Moran (University of Minnesota), Andrew Muir (Emory Children's Center), Jerry Palmer (University of Washington), Mark Peakman (King's College), Louis Philipson (University of Chicago), Philip Raskin (University of Texas Southwestern), Maria Redondo (Baylor Texas Children's Hospital), Henry Rodriguez (University of South Florida Diabetes and Endocrinology Center), William Russell (Vanderbilt Eskind Diabetes Clinic), Desmond A. Schatz (University of Florida), Jay M. Sosenko (University of Miami), Lisa Spain (National Institute of Diabetes and Digestive and Kidney Diseases [NIDDK]), John Wentworth (Walter & Eliza Hall Institute of Medical Research), Diane Wherrett (University of Toronto), Darrell M. Wilson (Stanford University), William Winter (University of Florida), Anette Ziegler (Technical University Munich).
Past Members: Mark Anderson (University of California, San Francisco), Peter Antinozzi (Wake Forest University), Richard Insel (Juvenile Diabetes Research Foundation [JDRF]), Thomas Kay (St. Vincent's Institute of Medical Research), Jennifer B. Marks (University of Miami), Alberto Pugliese (University of Miami), Bart Roep (Leiden University Medical Center), Jay S. Skyler (University of Miami), Jorma Toppari (Hospital District of Southwest Finland).

Executive Committee: Carla J. Greenbaum (Benaroya Research Institute), Jeffrey P. Krischer (University of South Florida), Lisa Spain (National Institute of Diabetes and Digestive and Kidney Diseases [NIDDK]).
Past Members: Katarzyna Bourcier (National Institute of Allergy and Infectious Diseases [NIAID]), Richard Insel (Juvenile Diabetes Research Foundation [JDRF]), John Ridge (National Institute of Allergy and Infectious Disease [NIAID]), Jay S. Skyler (University of Miami).

Chair's Office: Carla J. Greenbaum (Benaroya Research Institute), Lisa Rafkin (University of Miami), Jay M. Sosenko (University of Miami).
Past Members: Jay S. Skyler (University of Miami), Irene Santiago (University of Miami).

TrialNet Coordinating Center (University of South Florida): Jeffrey P. Krischer, Brian Bundy, Michael Abbondondolo, Timothy Adams, Ilma Asif, Jenna Bjellquist, Matthew Boonstra, Cristina Burroughs, Mario Cleves, David Cuthbertson, Meagan DeSalvatore, Christopher Eberhard, Steve Fiske, Julie Ford, Jennifer Garmeson, Susan Geyer, Brian Hays, Courtney Henderson, Martha Henry, Kathleen Heyman, Belinda Hsiao, Christina Karges, Beata-Gabriela Koziol, Lindsay Lane, Shu Liu, Jennifer Lloyd, Kristin Maddox, Jamie Malloy, Julie Martin, Cameron McNeill, Margaret Moore, Sarah Muller, Thuy Nguyen, Jodie Nunez, Ryan O'Donnell, Melissa Parker, MJ Pereyra, Amy Roberts, Kelly Sadler, Christine Sullivan, Roy Tamura, Elon Walker-Veras, Megan V. Warnock, Keith Wood, Rebecca Wood, Ping Xu, Vanessa Yanek, Kenneth Young.
Past Members: Darlene Amado, Amanda Kinderman, Ashley Leinbach, Jessica Miller, Nichole Reed, Tina Stavros.

National Institute of Diabetes and Digestive and Kidney Diseases [NIDDK]: Lisa Spain.

Data Safety and Monitoring Board: Emily Blumberg (University of Pennsylvania), Sean Aas (Georgetown University), Gerald Beck (Cleveland Clinic Foundation), Rose Gubitosi-Klug (Case Western Reserve University), Lori Laffel (Joslin Diabetes Center), Robert Vigersky (Medtronic), Dennis Wallace (Research Triangle Institute).
Past Members: David Brillon (Cornell University), Robert Veatch (Georgetown University).

Infectious Disease Safety Committee: Brett Loechelt (Children's National Medical Center), Lindsey Baden (Brigham and Women's Hospital), Peter Gottlieb (Barbara Davis Center for Childhood Diabetes), Michael Green (University of Pittsburgh), Adriana Weinberg (University of Colorado).

Collaborative Mechanistic Studies Panel: Peter A. Gottlieb (Barbara Davis Center for Childhood Diabetes), Mark Anderson (University of California, San Francisco), Mark A. Atkinson (University of Florida), Todd M. Brusko (University of Florida), Jane H. Buckner (Benaroya Research Institute), Carmella Evans-Molina (Indiana University), Kevan C. Herold (Yale University), Martin J. Hessner (Medical College of Wisconsin), Peter S. Linsley (Benaroya Research Institute), S. Alice Long (Benaroya Research Institute), Sarah Muller (University of Florida), David A. G. Skibinski (Benaroya Research Institute).
Past Members: Manuela Battaglia (San Raffaele University), Mark Peakman (King's College).

Laboratory Directors: Santica Marcovina (University of Washington), Jerry P. Palmer (University of Washington), Jay Tischfield (Rutgers University), Adriana Weinberg (University of Colorado), William Winter (University of Florida), Liping Yu (Barbara Davis Center for Childhood Diabetes).

TrialNet Clinical Network Hub (Benaroya Research Institute): Annie Shultz, Emily Batts, Arielle Pagryzinski, Mary Ramey, Meghan Tobin.
Past Members: Kristin Fitzpatrick, Randy Guerra, Melita Romasco, Christopher Webb.

Active Personnel at Sites Participating in the TN02, TN05, TN08, TN09, TN14, and/or TN19 protocol(s):

Barbara Davis Center for Childhood Diabetes, Aurora, Colorado: Peter Gottlieb, Kevin Deane, Jenna Lungaro, Aaron Michels, Andrea Steck, Christopher Striebich, Paul Wadwa, Ruthie Williamson.

Benaroya Research Institute, Seattle, Washington: Carla J. Greenbaum, Jane Buckner, Wei Hao, Sandra Lord, Marli McCulloch-Olson, Mary Ramey, Elaine Sachter, Jenna Snavely, Meghan Tobin, Corinna Tordillos, Dana VanBuecken.

Children's Hospital Los Angeles, Los Angeles, California: Roshanak Monzavi, Daniel Bisno, Lynda Fisher, Jennifer Raymond.

Columbia University, New York, New York: Robin Goland, Analia Alvarez, Magdalena Bogun, Rachelle Gandica, Natasha Leibel, Sarah Pollak, Barney Softness, Kristen Williams.

Greenville Health System Pediatric Endocrinology, Greenville, South Carolina: Elaine Apperson, James Amrhein, Lisa Looper, Andrew Smith, Lori Wise.

Indiana University, Indianapolis, Indiana: Linda DiMeglio, Carmella Evans-Molina, Emily Sims, Maria Spall, Stephanie Woerner.

Joslin Diabetes Center, Boston, Massachusetts: Jason Gaglia.

Stanford University, Stanford, California: Darrell M. Wilson, Nora Arrizon-Ruiz, Tandy Aye, Laura Bachrach, Karen Barahona, Bruce Buckingham, Trudy Esrey, Laura Nally.

University of California, San Francisco, California: Stephen E. Gitelman, Mark Anderson, Glenna Auerback, Jeanne Buchanan, Christine T. Ferrara, Karen Ko, Janet Lee, Srinath Sanda, Lorraine Stiehl, Christine Torok, Rebecca Wesch.

University of Florida, Gainesville, Florida: Michael J. Haller, Anastasia Albanese-O'Neill, Todd Brusko, Miriam Cintron, Jennifer Hosford, Laura M. Jacobsen, Henry Rohrs, Desmond A. Schatz, Janet Silverstein, Paula Towe.

University of Miami Diabetes Research Institute, Miami, Florida: David A. Baidal, Carlos Blaschke, Della Matheson, Janine Sanchez, Natalia Sanders-Branca, Jay S. Skyler, Jay M. Sosenko.

University of Minnesota, Minneapolis, Minnesota: Antoinette Moran, Janice Leschyshyn, Jennifer McVean, Brandon Nathan, Brittney Nelson, Beth Pappenfus, Jessica Ruedy, Anne Street, Muna Sunni, Darcy Weingartner.

University of Pittsburgh, Pittsburgh, Pennsylvania: Dorothy Becker, Kelli DeLallo, Ana Diaz, David Groscost, Mary Beth Klein, Ingrid Libman, Karen Riley.

University of South Florida Diabetes and Endocrinology Center, Tampa, Florida: Henry Rodriguez, Sureka Bollepalli, Rachel Brownstein, Emily Eyth, Danielle Henson, Michele Laine, Dorothy Shulman.

University of Texas Southwestern, Dallas, Texas: Phillip Raskin, Michael Phan.

Vanderbilt Eskind Diabetes Clinic, Nashville, Tennessee: William Russell, Faith Brendle, Anne Brown, Brenna Dixon, Justin Gregory, Dan Moore, James Thomas.

Walter and Eliza Hall Institute of Medical Research, Australia: Peter Colman, Marika Bjorasen.

Yale University, New Haven, Connecticut: Kevin Herold, Laurie Feldman, Jennifer Sherr, Robert Sherwin, William Tamborlane, Stuart Weinzimer.
